# Floral orientation affects outcross‐pollen deposition in buzz‐pollinated flowers with bilateral symmetry

**DOI:** 10.1002/ajb2.16078

**Published:** 2022-10-17

**Authors:** Lucy Nevard, Mario Vallejo‐Marín

**Affiliations:** ^1^ Biological & Environmental Sciences University of Stirling Stirling UK FK9 4LA

**Keywords:** buzz pollination, floral orientation, floral symmetry, mating systems, pollen deposition, Solanaceae, *Solanum*

## Abstract

**Premise:**

Floral orientation is central to plant–pollinator interactions and is commonly associated with floral symmetry. Bilaterally symmetrical flowers are often oriented horizontally for optimal pollinator positioning and pollen transfer efficiency, while the orientation of radially symmetrical flowers is variable. Buzz‐pollinated species (pollinated by vibration‐producing bees) include bilateral, horizontally oriented flowers, and radial, pendant flowers. The effect of floral orientation on pollen transfer has never been tested in buzz‐pollinated species.

**Methods:**

Here, we examined the effect of floral orientation on bumblebee‐mediated pollen deposition in three buzz‐pollinated *Solanum* species with different floral symmetry and natural orientations: *S. lycopersicum* and *S. seaforthianum* (radial, pendant), and *S. rostratum* (bilateral, horizontal). We tested whether orientation affects total stigmatic pollen deposition (both self and outcross pollen) when all flowers have the same orientation (either pendant or horizontal). In a second experiment, we evaluated whether different orientations of donor and recipient flowers affects the receipt of outcross pollen by *S. rostratum*.

**Results:**

For the three *Solanum* species studied, there was no effect of floral orientation on total pollen deposition (both self and outcross) when flowers shared the same orientation. In contrast, in our experiment with *S. rostratum*, we found that pendant flowers received fewer outcross‐pollen grains when paired with pendant donors.

**Conclusions:**

We suggest that floral orientation influences the quality of pollen transferred, with more outcross pollen transferred to horizontally oriented recipients in the bilaterally symmetrical *S. rostratum*. Whether other bilaterally symmetrical, buzz‐pollinated flowers also benefit from increased cross‐pollination when presented horizontally remains to be established.

Floral orientation, the position of the corolla's main axis relative to the horizontal, plays an important role in plant reproductive success and in mediating plant–pollinator interactions (Stebbins, 1974; Fenster et al., 2009; Armbruster and Muchhala, 2020). A pendant orientation might protect pollen grains and nectar from rainfall (Huang et al., 2002; Aizen, 2003; Wang et al., 2010; Lin and Forrest, 2017; Nakata et al., 2022), heat (Haverkamp et al., 2019), or favor certain types of floral visitors (Ushimaru et al., 2009; Makino and Thomson, 2012; Campbell et al., 2016; Haverkamp et al., 2019). Meanwhile, a horizontal or vertical orientation might make flowers more attractive to pollinators or easier to access (Haverkamp et al., 2019; Nakata et al., 2022). Crucially, a horizontal orientation in bilaterally symmetrical flowers is understood to promote precise pollinator positioning and improve pollen transfer, relative to pendant flowers (Giurfa et al., 1999; Fenster et al., 2009; Armbruster and Muchhala, 2020; Stewart et al., 2022). According to the pollen position hypothesis (Neal et al., 1998), bilateral flowers constrain the position of the bee pollinator on the flower and promote consistency in the site of contact between the pollinator body and the flower's sexual organs, impacting both pollen removal and deposition (Neal et al., 1998; Giurfa et al., 1999; Ushimaru et al., 2009; Armbruster et al., 2014; Culbert and Forrest, 2016; Armbruster and Muchhala, 2020). In radially symmetrical flowers, pollen placement is thought to be less precise, pollinator positioning less crucial, and floral orientation under fewer selective constraints (Naghiloo et al., 2020; Stewart et al., 2022). In some taxa, radial symmetry is associated with a vertical orientation (Stewart et al., 2022), but they can also benefit from the increased attractiveness of a horizontal orientation (Nakata et al., 2022). In contrast, bilateral flowers are consistently associated with horizontal orientations across angiosperms (Giurfa et al., 1999; Ushimaru et al., 2009; Naghiloo et al., 2020; Stewart et al., 2022). The combination of bilateral symmetry and horizontal orientation further restricts pollinator positioning, limiting the angles from which a pollinator can approach the flower and increasing the precision of pollen placement (Fenster et al., 2009; Naghiloo et al., 2020). Selection for maintaining a horizontal orientation in bilateral flowers to achieve optimal floral function is thought to explain why they are more likely than radial flowers to restore their horizontal orientations after being experimentally manipulated (Armbruster and Muchhala, 2020).

The drivers of floral orientation are likely to vary across taxa according to pollination system and floral morphology, and here we investigated the effect of floral orientation in buzz‐pollinated plants. Buzz‐pollinated plants are those that rely on bee vibrations for pollen release and include about 20,000 species with diverse floral orientations commonly including pendant flowers and, in some cases, horizontally oriented flowers (Macior, 1964; Buchmann, 1983; Kawai and Kudo, 2009). Buzz‐pollinated flowers are often nectarless and have pollen grains enclosed in tubular anthers with small apical pores (poricidal anthers) (Buchmann, 1983; Vallejo‐Marín et al., 2010; Brito and Sazima, 2012), suggesting that protection against rain or heat might not be a strong driver of floral orientation. Moreover, pollen ejection in buzz‐pollinated flowers with poricidal anthers is probably unaffected by floral orientation (M. Vallejo‐Marín, personal observations; it has not been rigorously tested), and bee pollinators successfully remove pollen in flowers with different orientations (Corbet et al., 1988; Papaj et al., 2017; Rosi‐Denadai et al., 2020). Pollinator preferences might not be a strong driver of floral orientation in buzz‐pollinated flowers, which can only be efficiently pollinated by bees (but see Buchmann et al., 1977; Vallejo‐Marín and Vallejo, 2021). Although some bees (including bumblebees, *Bombus* spp.) tend to prefer vertical or horizontal to pendant flowers (Makino and Thomson, 2012; Nakata et al., 2022), even when flowers are naturally pendant (Prokop et al., 2020), bees will readily visit all orientations and can handle pendant flowers more effectively than other insects can (Wang et al., 2014; Haverkamp et al., 2019; Prokop et al., 2020). A pendant orientation in buzz‐pollinated flowers may therefore serve to exclude unwanted or illegitimate visitors, although this possibility has not been thoroughly investigated. In contrast, a horizontal orientation might play a key role in pollen transfer, particularly in bilaterally symmetrical buzz‐pollinated flowers that require the precise positioning of pollinators during visitation. Several aspects of buzz‐pollinated flowers may also mediate the effect of orientation, such as their unique method of pollen ejection, their specific floral morphologies, and the behavior of buzzing bees compared to other foraging behaviors. To our knowledge, the effect of floral orientation on pollen transfer in buzz‐pollinated flowers has not previously been tested and is the main focus of our study.

Here, we investigated the effect of floral orientation on stigmatic pollen deposition using three buzz‐pollinated, nectarless species of *Solanum* L. (Solanaceae) with different floral symmetries and orientations: *Solanum lycopersicum* [sect. *Lycopersicon* (Mill.) Wettst.; tomato]*, S. rostratum* Dunal (sect. *Androceras* Whalen), and *S. seaforthianum* Andrews (sect. *Dulcamara* sensu Nee [1999]; Dulcamaroid clade [Weese and Bohs, 2007; Gagnon et al., 2022]). Both *S. lycopersicum* and *S. seaforthianum* are radially symmetrical with anthers surrounding the stigma and are naturally pendant (Figure 1A, B). *Solanum rostratum* is bilaterally symmetrical, heterantherous (two or more morphologically distinct anthers within a flower), enantiostylous (two mirror‐image floral morphs that alternate along an inflorescence), horizontally oriented, and outcrossed (Jesson and Barrett, 2002; Vallejo‐Marín et al., 2010, 2013). We used experimental arrays with flowers arranged in different orientations and exposed to bumblebee visitors (*Bombus terrestris*) to address the following questions: (1) Does floral orientation (pendant vs. horizontal) affect total pollen deposition (self and outcross) in each of these three species? In bilateral *S. rostratum* flowers, we expected that horizontal flowers would have higher total pollen deposition than pendant flowers in the *S. rostratum*, due to their requirement for precise bee positioning on the flower and the likelihood that bees adopt different positions on each orientation (Solís‐Montero and Vallejo‐Marín, 2017). In the radial flowers, we predicted little effect of orientation on pollen deposition (Armbruster and Muchhala, 2020). (2) In a naturally horizontal flower, does the correspondence between donor and recipient orientation affect outcross‐pollen deposition? We predicted that a mismatch between donor and recipient orientation will reduce outcross‐pollen deposition in *S. rostratum* due to a discrepancy between pollen placement by the anthers and contact with the stigma (Solís‐Montero and Vallejo‐Marín, 2017).

**Figure 1:**
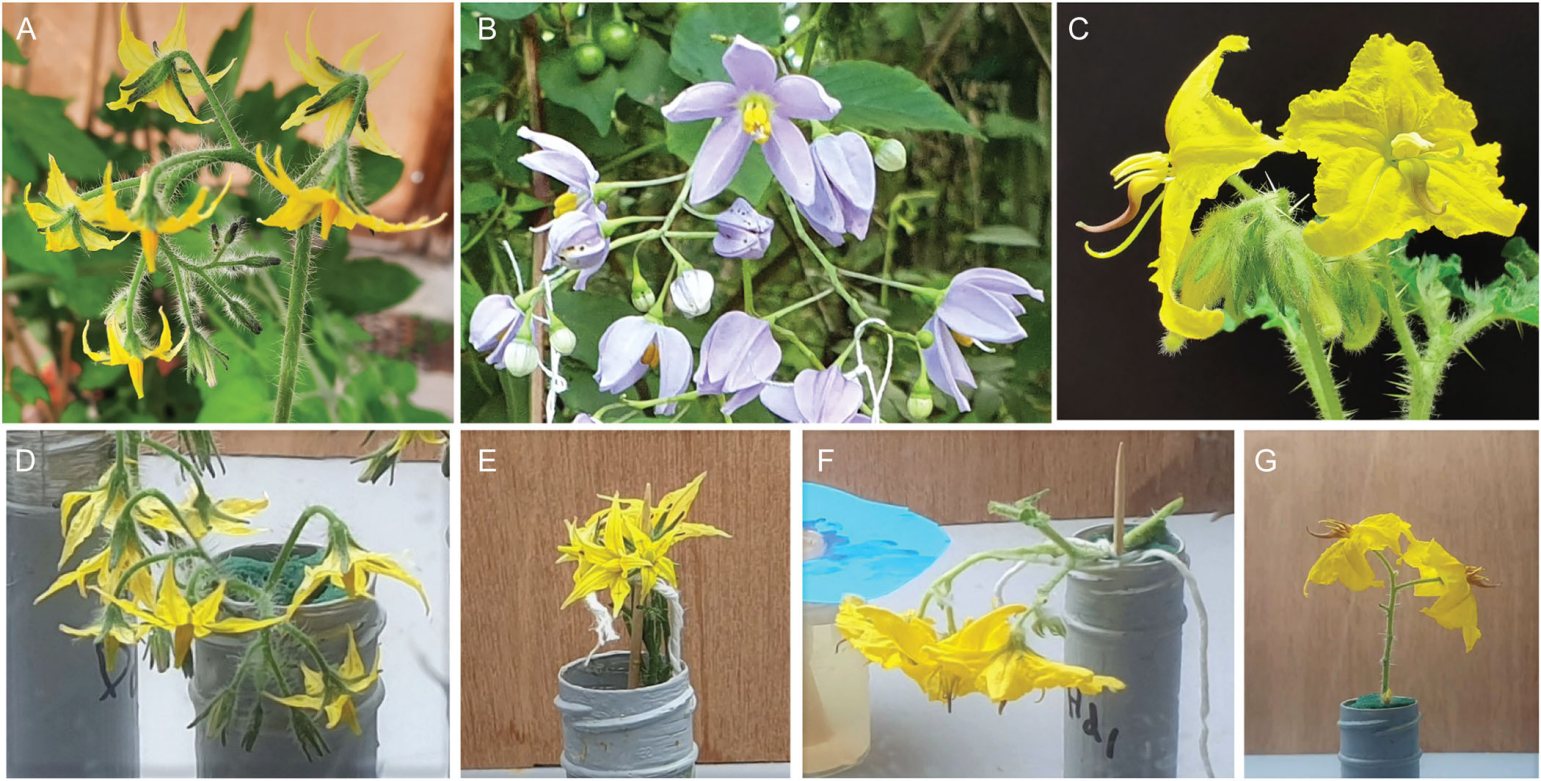
Three buzz‐pollinated *Solanum* species used in pollen deposition experiments and the manipulation of their orientation: (A) *S. lycopersicum* (pendant, experiment one), (B) *S. seaforthianum* (pendant, experiment one), (C) *S. rostratum* (horizontal, experiments one and two), (D) pendant *S. lycopersicum*, (E) horizontal *S. lycopersicum*, (F) pendant *S. rostratum*, (G) horizontal *S. rostratum*.

## MATERIALS AND METHODS

### Study system

*Solanum* (Solanaceae) contains around 1400 species (Särkinen et al., 2013) of mostly buzz‐pollinated flowers with floral orientations ranging from pendant (e.g., *Solanum dulcamara*, *S. lycopersicum*) to horizontal (e.g., *S. citrullifolium*, *S. rostratum*) (Vallejo‐Marín et al., 2010) and occasionally almost vertical (anthers pointing upward; e.g., in some flowers of *S. houstonii*) (Knapp et al., 2017). The three *Solanum* species studied here differ in floral orientation and symmetry. Flowers of *S. lycopersicum* are bright yellow, radially symmetrical, with a tightly fused anther cone around the central stigma (Glover et al., 2004) (Figure 1A). The natural orientation of *S. lycopersicum* flowers is pendant, i.e., downward facing, with the flower's main axis perpendicular to the ground. *S. lycopersicum* is self‐compatible but bee pollination improves tomato yield (Cooley and Vallejo‐Marín, 2021). For our experiments, we used the cherry tomato F1 hybrid variety Sweet Million (Premier Seeds Direct, Salisbury, UK). The flowers of this variety of cherry tomato are relatively small, with an average corolla width of approximately 2.2 mm, and the stigma protruding just above the anther cone. The second species, *S. seaforthianum* is a vine native to tropical South America and cultivated or invasive in many parts of the world (Jagatheeswari, 2014) (see Appendix S1 for accession details). Flowers have relatively large (average width of 23 mm), radially symmetrical violet corollas, with five yellow stamens, unfused but loosely grouped around an exerted style (average length of 10.1 mm) and one stamen slightly longer than the other four (average lengths of 8.2 mm and 6.7 mm, respectively) (Figure 1B). They are naturally pendant in orientation, with the flower's main axis perpendicular to the ground. *Solanum seaforthianum* is self‐compatible, but its mating system is unknown, and it is visited by bees, including *Eulaema* sp. (L. Nevard, personal observations).

*Solanum rostratum*, is native to North America (Whalen, 1978; Zhao et al., 2013) and invasive in other parts of the world including China (Zhao et al., 2013) (Appendix S1 for accession details). Flowers of *S. rostratum* are relatively large (width 28 mm), yellow, bilaterally symmetrical, enantiostylous, and heterantherous (Todd, 1882; Bowers, 1975; Jesson and Barrett, 2002; Vallejo‐Marín et al., 2010) (Figure 1C). The single pollinating anther is usually darker in color, longer, S‐shaped, and the stamen has an average length of 14.3 mm and contributes proportionally more to pollination (Vallejo‐Marín et al., 2009). The four remaining yellow anthers (feeding anthers) with stamens averaging 9.7 mm long, are shorter, straight, and more attractive to visiting bees while contributing proportionally less to pollination (Vallejo‐Marín et al., 2009). The style and pollinating anther are deflected to opposite sides within a flower, and two floral enantiomorphs alternate in the same inflorescence (Jesson and Barrett, 2002). The natural orientation of these flowers is horizontal, with the main axis parallel to the ground (Whalen, 1978). In heterantherous, enantiostylous species, effective outcross‐pollen transfer likely relies on bees contacting both the anther and the stigma at equivalent locations in the pollinator's body (Jesson and Barrett, 2002; Solís‐Montero and Vallejo‐Marín, 2017). Pollinator positioning is therefore probably key in these systems and is likely aided by a horizontal orientation restricting the angle from which the bee can approach and land. Flowers with heteranthery, including those seen in *Solanum* section *Androceras*, are usually horizontally oriented, while radially symmetrical flowers with anther cones are often pendant or between pendant and horizontal (Bohs et al., 2007). *Solanum rostratum* is self‐compatible, but predominantly outcrossing (outcrossing rate, *t* = 0.70) (Vallejo‐Marín et al., 2013; Zhang et al., 2017). Wild populations are pollinated by a variety of medium and large buzzing bee species, and flowers are also readily buzzed by captive bumblebees (Bowers, 1975; De Luca et al., 2013; Solís‐Montero et al., 2015; Arroyo‐Correa et al., 2019). Usually, medium to large bees grasp the feeding anthers with their mandibles and contact the pollinating anther with the abdomen (Vallejo‐Marín et al., 2009; Solís‐Montero et al., 2015).

### Plant growth

Plants were grown at the University of Stirling in the summer of 2020. Seeds of *S. rostratum* and *S. seaforthianum* were collected from wild populations in Mexico in 2010 and 2019, respectively. Seeds of *S. lycopersicum* were purchased from Premier Seeds Direct, Salisbury, UK. Seeds were treated with 1000 ppm solution of gibberellic acid (GA3: Sigma‐Aldrich, Dorset, UK) to induce germination as described by Vallejo‐Marín et al. (2014). Seeds were planted in modular seed compost (William Sinclair Horticulture, Lincoln, UK) in seed trays and kept in growth chambers for 4 weeks with 18 h light/6 h dark at 28°C with 52% relative humidity. Seedlings were transplanted to 1.5‐L pots in a 4:1 mix of all‐purpose growing medium and perlite (William Sinclair Horticulture). Plants were then kept in a pollinator‐proof glasshouse, and fertilized weekly with Tomorite (Levington, Surrey, UK). Glasshouses were supplemented with artificial fluorescent lighting (16 h), and supplemental heating was provided if temperature dropped below 14°C.

### Bees

Experiments were performed using workers from two colonies of commercially obtained buff‐tailed bumblebee (*Bombus terrestris audax*) (Biobest, supplied by Agralan, Swindon, UK). Colonies were provisioned with Biogluc sucrose solution underneath the colony (Biobest Group NV, Westerlo, Belgium) and ground honeybee‐collected pollen (Agralan). Room temperature was 18–20°C.

### Experimental set‐up

Two bumblebee colonies were attached to a grey‐painted flight arena (60 × 60 × 37 cm) fitted with a UV‐transparent acrylic top and illuminated from above with an LED light panel (59.5 × 59.5 cm, 48 W Daylight; Opus Lighting Technology, Birmingham, UK). Before the experiments, bees gained experience freely foraging on *S. rostratum* flowers in the flight arena. Flowers were collected from the glasshouses in the morning of the experiments and presented in individual tubes filled with floral foam (OASIS Floral Products, Washington, UK). A nectar feeder containing 1 M sucrose solution was also provided in the arena. In the experiments, we used two floral orientations for each species: pendant and horizontal. In the pendant treatment, the main floral axis is perpendicular to the ground, while in the horizontal treatment, it is parallel to the ground. All flowers of our *S. lycopersicum* and *S. seaforthianum* plants are naturally pendant, while the majority of *S. rostratum* flowers are naturally horizontal (90°), and some are between horizontal and vertical (up to 135°). We used horizontal flowers for our experiments. We used toothpicks and string to arrange flowers differently to their natural orientation (Figure 1D–G). A naturally pendant flower stem was tied to a toothpick inside the floral foam tube to keep the flower horizontal. A naturally horizontal flower was tied to a toothpick that was positioned perpendicular to the tube. Flowers were positioned to be at a consistent height from the floor of the arena, regardless of orientation treatment. Flowers came from 8–10 individual plants for *S. lycopersicum*, 15–20 plants for *S. rostratum*, and 1 plant for *S. seaforthianum*.

### Effect of floral orientation on total pollen deposition (self and outcross)

In the first experiment, we assessed the effect of floral orientation (horizontal or pendant) on total stigmatic pollen deposition; i.e., all flowers were intact, and pollen could be either self or outcross. We studied three plant species with differing natural orientations: *S. lycopersicum*, *S. rostratum*, and *S. seaforthianum*. In each trial, bees were presented with between 15–35 flowers of the same species, depending on flower availability. Within a trial, all flowers were presented in the same orientation within the arena, either horizontal or pendant. Depending on availability, between 10 and 20 bees were released to freely forage on flowers for at least 60 min and up to a maximum of 90 min, unless foraging activity had clearly ceased by this time. After the trial had ended, styles were carefully removed from all flowers for stigma pollen counting.

### Effect of complementarity of floral orientation on outcross pollen

In the second experiment, we assessed the effect of the correspondence between donor and recipient orientation on pollen transfer in *S. rostratum*. This species was chosen because it has a high outcrossing rate (*t* = 0.70; Vallejo‐Marín et al., 2013; Zhang et al., 2017) and heterantherous and enantiostylous flowers, in which orientation is probably required for pollination (Vallejo‐Marín et al., 2009) and outcrossing (Jesson and Barrett, 2002). In our experiment, 75% of trials consisted of 12 flowers, in which four were pollen donors and eight were pollen recipients. As flowers became more limited, later in the experiment, 25% of trials consisted of nine flowers, in which three were pollen donors and six were recipients. We removed the styles from donors and glued shut the anthers of recipients with silicone glue. There were four combinations of donor–recipient orientations: horizontal–horizontal (H‐H), pendant–horizontal (P‐H), horizontal–pendant (H‐P), and pendant–pendant (P‐P). Each trial contained one of these combination treatments. Flowers were arranged randomly in the arena and between four and nine bees were released to freely forage for 30 min. After the trial had ended, styles were carefully removed from all flowers for stigma pollen counting.

### Pollen counting

Stigmas were removed and mounted on microscope slides with melted fuchsin jelly for pollen staining (Kearns and Inouye, 1993). Total pollen loads on stigmas were counted under a binocular microscope (Olympus CX31, Tokyo, Japan) using 400× magnification.

### Statistical analyses

We evaluated the effect of floral orientation on pollen loads in each stigma using generalized linear mixed models (GLMM) with a negative binomial error distribution and a log link function, using the MASS package (Venables and Ripley, 2002). A negative binomial distribution was chosen to account for high levels of dispersion in the data (White and Bennetts, 1996). In all models, the response variable was pollen load in each stigma. For experiment one, a first model was fitted with an interaction term between floral orientation and plant species, and trial number was used as a random effect. A second model was fitted without the interaction term. Akaike's information criterion (AIC in the stats package; R Core Team, 2021) was used to compare models. For experiment two, a first model was fitted with an interaction between donor orientation and recipient orientation, and trial number was used as a random effect. A second model was fitted without the interaction term. AIC was used to compare models. The DHARMa package was used to produce residual diagnostic tests for each model (Hartig, 2019). Statistical significance of the main effects was assessed using the car package (ANOVA, Type II sums of squares for models with no interaction term and Type III for those with an interaction term) (Weisberg, Fox, 2019). All statistical analyses were performed in R 4.0.2 (R Core Team, 2021).

### Ethics

Bumblebee experiments were approved by the Animal Welfare and Ethical Review Board at the University of Stirling.

## RESULTS

### Effect of floral orientation on total pollen deposition (self and outcross)

**Figure 2:**
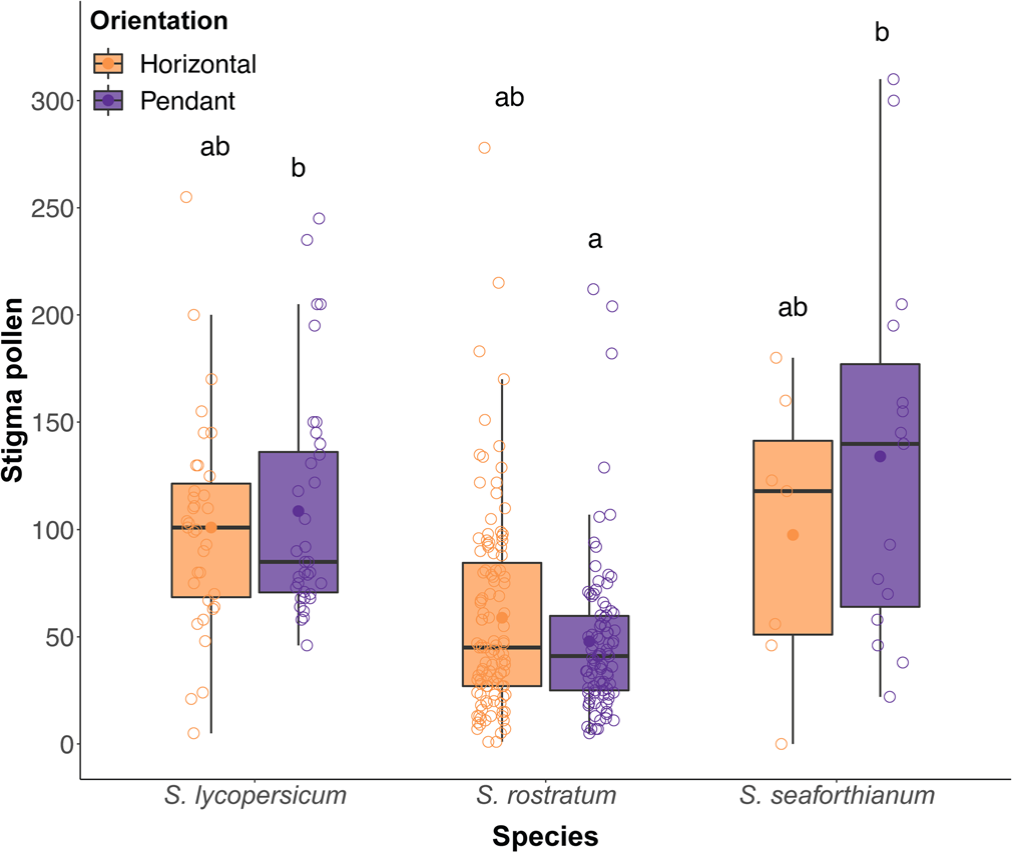
The effect of orientation on total (self and outcross) pollen deposition for three *Solanum* species: *S. lycopersicum*, *S. seaforthianum* and *S. rostratum*. Stigma pollen counts for each species are grouped by treatment (horizontal or pendant). *S. lycopersicum* (*N* = 35 in horizontal, *N* = 36 in pendant), *S. seaforthianum* (*N* = 20 in horizontal, *N* = 35 in pendant), *S. rostratum* (*N* = 154 in horizontal, *N* = 96 in pendant). Lowercase letters indicate pairwise comparisons between species–treatment combinations and different letters indicate significant pairwise differences (Tukey's test).

Across the three *Solanum* species, we completed 19 trials (423 flowers total, from which 334 styles were collected and prepared for pollen counting; Table 1). We found no interaction between floral orientation and plant species on pollen deposition (AIC), and thus the interaction term was dropped from the model. In the simpler model, we found significant differences in pollen deposition among species (estimate for *S. rostratum*: –0.74, for *S. seaforthianum*: 0.12, –0.74, *P* < 0.01; Table 2), with fewer pollen grains deposited on *S. rostratum* stigmas than on *S. lycopersicum* or *S. seaforthianum* stigmas (Figure 2, Table 2). However, we found no evidence to suggest that floral orientation had an overall effect on pollen deposition (self and outcross) when all flowers in the array have the same orientation; Figure 2, Table 2).

**Table 1:** Total pollen deposition (number of pollen grains) in floral arrays consisting of flowers of a single species arranged in the same floral orientation (either pendant or horizontal). Stigma pollen load means, standard errors, ranges, and sample sizes grouped by species. N = number of flowers analyzed.

	Horizontal			Pendant		
Species	Mean ± SE	Range	*N*	Mean ± SE	Range	*N*
***S. lycopersicum***	101 ± 8.3	5–250	35	108.5 ± 8.8	46–240	36
***S. rostratum***	59.7 ± 3.9	0–278	154	47.9 ± 3.8	5–212	96
***S. seaforthianum***	97.6 ± 24.7	0–180	20	134.5 ± 23.2	22–310	35

**Table 2:** Generalized linear mixed model (negative binomial error distribution) fitted for experiment one: total pollen deposition in three species. Model is fitted with stigmatic pollen count as the response variable, with orientation and species as fixed effects, and trial number as a random effect. *P*‐value of fixed effects calculated using Type II sums of squares. Sample sizes: *S. lycopersicum* (71), *S. rostratum* (241), *S. seaforthianum* (22).

Experiment one	Estimate	SE	*P*
Intercept (*S. lycopersicum*; horizontal)	4.67	0.48	**<0.001**
Orientation (pendant)	–0.03	0.16	0.83
Species			**<0.01**
*S. rostratum*	–0.74	0.2	
*S. seaforthianum*	0.12	0.3	

### Effect of donor and recipient floral orientation on outcross‐pollen deposition in *S. rostratum*

**Figure 3:**
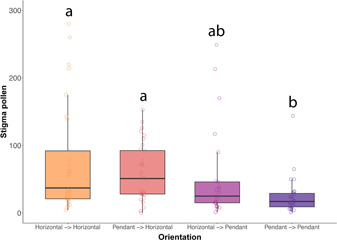
The effect of complementarity of floral orientation on outcross pollen in *S. rostratum*. Stigma pollen counts for *S. rostratum*, grouped by orientation treatment (horizontal–horizontal (*N* = 29), pendant–horizontal (*N* = 25), horizontal–pendant (*N* = 32), pendant–pendant (*N* = 27)). Lowercase letters indicate pairwise comparisons between orientation treatments and different letters indicate significant pairwise differences (Tukey's test).

In our second experiment with *S. rostratum*, we completed 16 trials using 180 flowers (60 donors and 120 recipients), of which 119 recipient stigmas were collected for pollen counting (donor–recipient orientation: stigma number, H‐H: 30, H‐P: 32, P‐H: 29; P‐P: 28;), and six stigmas were excluded from analysis due to issues with mounting or pollen visibility (Table 3). We found a significant effect of floral orientation on outcross‐pollen receipt (*P* < 0.01, Table 4). In particular, the pendant–pendant combination had a significantly lower pollen receipt than either the horizontal–horizontal (estimate: –1.12, *P* < 0.001) or pendant–horizontal (estimate: –0.82, *P* < 0.05) combinations according to a pairwise honest significant difference (Tukey) test (Figure 3 and Table 3). The horizontal–pendant treatment had relatively low levels of pollen deposition, but the differences with other treatments were not statistically different (Tables 3 and 4; Figure 3).

**Table 3:** Experiment two: outcross‐pollen deposition. Stigma pollen load means, standard errors, ranges, and sample sizes for S. rostratum, grouped by orientation treatment.

Orientation	Mean ± SE	Range	*N*
Horizontal–horizontal	76.5 ± 15	4–280	30
Pendant–horizontal	58.9 ± 7.5	0–153	32
Horizontal–pendant	51.4 ± 13.3	1–249	29
Pendant–pendant	24.9 ± 5.5	1–144	28

**Orientation**	**Mean** ± **SE**	**Range**	***N***
Horizontal–horizontal	76.5 ± 15	4–280	30
Pendant–horizontal	58.9 ± 7.5	0–153	32
Horizontal–pendant	51.4 ± 13.3	1–249	29
Pendant–pendant	24.9 ± 5.5	1–144	28

**Table 4:** Generalized linear mixed model (negative binomial error distribution) fitted for experiment two: outcross‐pollen deposition. Fitted with stigmatic pollen count as the response variable and orientation (donor–recipient) as a fixed effect. P‐values calculated using Type II sums of squares. Sample size: 113.

Experiment two	Estimate	SE	*P*
Orientation			**<0.01**
Pendant–horizontal	–0.3	0.29	
Horizontal–pendant	–0.46	0.31	
Pendant–pendant	–1.12	0.3	

## DISCUSSION

Our results suggest that floral orientation does not affect the combined transfer of total (self plus outcross) pollen in three *Solanum* species, two of which have radially symmetric flowers. In contrast, floral orientation does impact the transfer of outcross pollen in the morphologically complex, bilaterally symmetric flowers of *S. rostratum*, as expected. However, outcross‐pollen receipt was only significantly lower when both donor and recipient flowers were pendant, rather than when there was a mismatch in orientation between donor and recipient. Together, these results suggest that the horizontal orientation of bilaterally symmetric, buzz‐pollinated flowers is particularly important in mediating the quality of pollen received (outcross pollen) rather than the total quantity of pollen delivery (self and outcross pollen).

### Consequences of floral orientation in radially symmetric flowers

**Figure 4:**
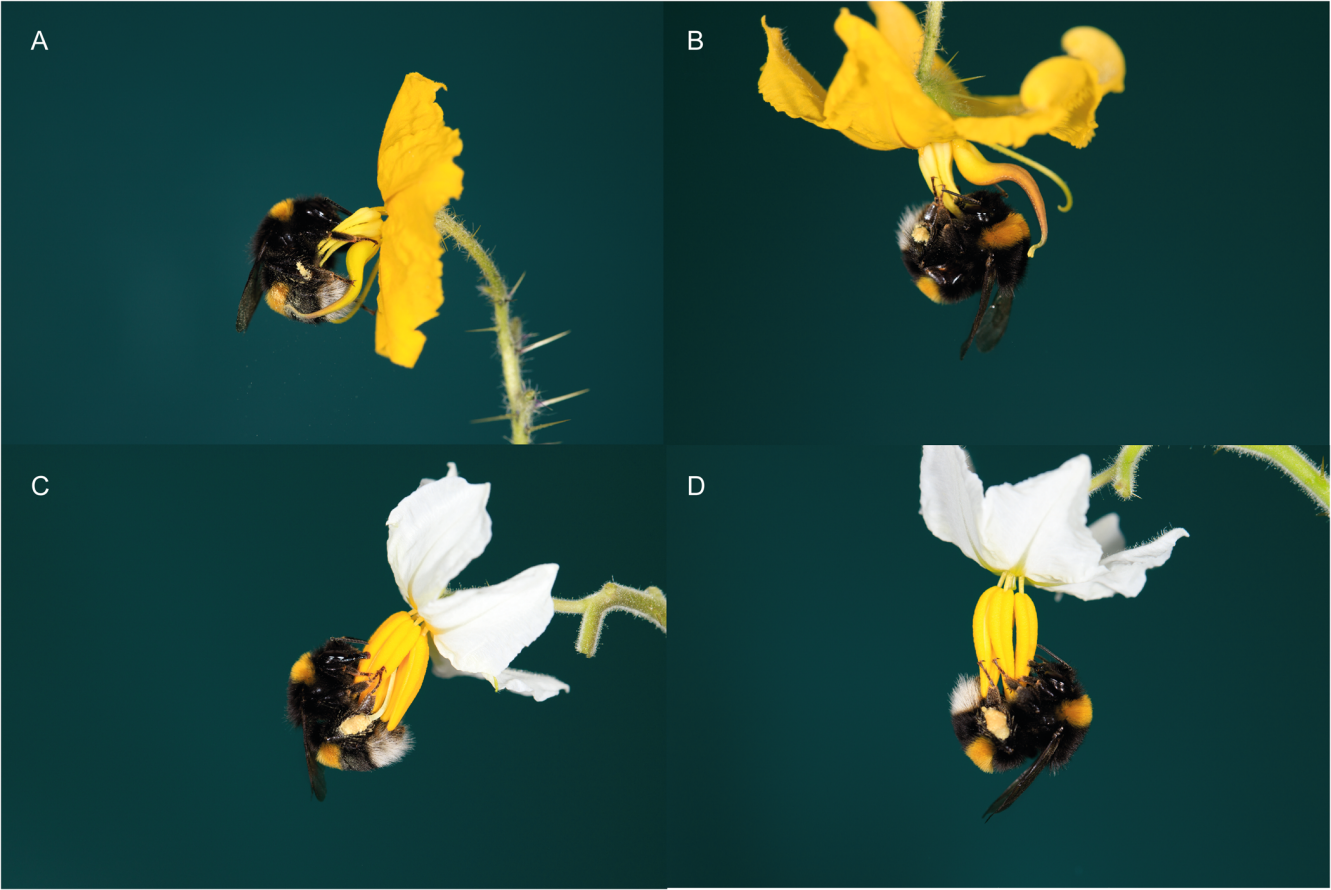
The position of a foraging bumblebee (*Bombus terrestris audax*) on bilateral and radial *Solanum* flowers in two orientations. (A) Horizontal *S. rostratum*, (B) pendant *S. rostratum*, (C) horizontal *S. sisymbriifolium*, (D) pendant *S. sisymbriifolium*. Notice that *S. sisymbriifolium* is used here as an example of radially symmetric *Solanum* flowers but was not used in the present study.

As expected, pollen deposition (self and outcross) in the radially symmetrical, buzz‐pollinated flowers of *S. lycopersicum* and *S. seaforthianum* was not affected by our manipulation of their orientation from pendant to horizontal as shown in other non‐buzz‐pollinated systems (Armbruster and Muchhala, 2020; Stewart et al., 2022). Radially symmetric buzz‐pollinated flowers may be insensitive to orientation as pollen can be ejected from anthers in any orientation, and the bee is likely to contact the stigma and anthers in similar places regardless of the direction of floral approach. In fact, buzz‐pollinating bees visiting flowers of *S. dulcamara* and *S. sisymbriifolium* regularly buzz and rotate around the anther cone during visitation (M. Vallejo‐Marín, personal observations), suggesting that the flower indeed can be manipulated and buzzed from different angles. In buzz‐pollinated flowers, where the stigma is presented centrally and protrudes from the anther cone, as in the radially symmetric species studied here, the bee is likely to contact the anther pores and stigma regardless of its direction of approach, suggesting that floral orientation in this type of flowers should have little effect on pollen transfer (Figure 4C, D). Nevertheless, orientation may still be important in other radially symmetric flowers or for reasons unrelated to pollinator visitation. For example, in nectar‐producing *Platycodon grandiflorus* (Campanulaceae), experimentally switching floral orientation from horizontal to upward increases rain damage to anthers, and switching to downward orientation reduces pollen receipt compared to the natural, mostly horizontal orientation in this species (Nakata et al., 2022).

### Effect of floral orientation in complex, bilaterally symmetric flowers

We found that in the bilaterally symmetric, morphologically complex flowers of *S. rostratum*, which are naturally presented in a horizontal plane, shifts in floral orientation affects outcross‐pollen deposition. We hypothesize that reduced outcross‐pollen transfer in the pendant–pendant experimental treatment arises from increased variation in the alignment of flower and bee during visitation to multiple flowers in pendant orientations compared to horizontal ones. Horizontal flowers permit pollinators to land only from a limited number of directions (Ushimaru and Hyodo, 2005; Naghiloo et al., 2020), promoting constancy in position between flowers (Ushimaru et al., 2009), as between donors and recipients in our experiment, and allowing more efficient pollen transfer. Pendant flowers allow approaches from all directions equally (Ushimaru and Hyodo, 2005; Wang et al., 2014), potentially reducing pollen transfer efficiency. In bilateral flowers, the direction of approach affects the proportion of legitimate landings (bee touching anthers and stigma), and the amount of pollen both collected and deposited by pollinators (Ushimaru and Hyodo, 2005; Ushimaru et al., 2009).

The heterantherous, enantiostylyous flowers of *S. rostratum* may require consistent and stereotypical alignment between the bee's body and the flower's sexual organs as is hypothesized to occur in bilateral flowers in general (Fenster et al., 2009; Armbruster and Muchhala, 2020; Stewart et al., 2022). If pendant flowers cause increased variation in the location of contact between anthers and stigma and the pollinator's body, as found in a study of 33 bee‐pollinated plants (Stewart et al., 2022), then outcross‐pollen transfer efficiency might be lower. A recent study on the enantiostylous *Wachendorfia paniculata* (Haemodoraceae), showed that stigma position is “fine‐tuned” to contact specific sites on the pollinator's body (Minnaar and Anderson, 2021). Orientation might also mediate the division‐of‐labor function of heteranthery (Papaj et al., 2017). Effective division of labor relies on pollen from the large pollinating anther being transferred effectively, often via “safe sites” on the bee's body that cannot be easily groomed (Vallejo‐Marín et al., 2009; Koch et al., 2017; Tong and Huang, 2018). It is possible that either the relative proportions of pollinating and feeding pollen transferred, the total amount of pollen on the bee's body available after grooming, and/or the place in which anthers and stigmas of flowers of different floral enantiomorphs contact the pollinator, depends on floral orientation. These possibilities are supported by our bee observations, which indicate that contact between the single pollinating anther and the bee may be most affected by floral orientation, while contact between the four central feeding anthers and the bee might be relatively reliable despite manipulations of orientation (Figure 4A, B).

The reduction in outcross‐pollen grains received in pendant flowers of *S. rostratum* observed in the second experiment, is not reflected in our analysis of the effect of orientation on total pollen deposition (self and outcross) from our first experiment. We hypothesize that although total pollen deposition remains the same in this species regardless of floral orientation, the relative composition of self vs. outcross pollen changes. For this difference in proportions to be apparent, increased self‐pollen transfer should have compensated for reductions of outcross‐pollen transfer in pendant orientations. Unfortunately, we could not distinguish self vs. outcross pollen deposited on the stigmas in the first experiment, and this hypothesis remains to be tested.

### Pollinator behavior and floral orientation

The effect of orientation on pollen transfer is also probably mediated by changes in pollinator behavior, although we did not explicitly test this in our study. Experimentally created pendant flowers of *S. rostratum* may be more difficult to manipulate by buzz pollinating because of their dimorphic anthers (Bowers, 1975; Figure 4), potentially changing their pollen collecting behavior and/or increasing the amount of time bees spend on pendant flowers after landing compared to horizontal flowers of this type (Laverty, 1980; Muth et al., 2015). Longer visits may lead to increased self‐pollen deposition (Kudo, 2003). Moreover, the amount of outcross pollen received in pendant flowers may decrease due to receiving fewer visits, if, for example, bees avoid visiting more difficult to manipulate pendant flowers. Studies in other buzz‐pollinated plants with horizontally oriented flowers show that differences in pollen collection behavior alter the placement of removed pollen on the bee body, impacting its deposition onto stigmas, and the amount of self‐pollen deposited relative to outcross pollen (Russell et al., 2021).

Bumblebee preferences for specific orientations may also play a role in our experiments. *Bombus impatiens* prefers visiting vertical flowers over pendant flowers (Makino and Thomson, 2012). In field experiments using bilaterally symmetrical flowers, bumblebees have been found to preferentially visit horizontally oriented (unmanipulated) flowers over either pendant or vertical flowers (Ushimaru and Hyodo, 2005), which can result in more pollen removed and higher seed set in horizontal flowers (Wang et al., 2014). However, this preference is not consistently found. Nakata et al. (2022) found no difference in visitation rates between horizontal and pendant flowers of *Platycodon grandiflorus*, and Huang et al. (2002) found no pollinator preference in *Pulsatilla cernua* (Ranunculaceae) flowers, which change from horizontal to pendant throughout their life cycle. In our experiments, when there is a choice between horizontal and pendant flowers (for example, when donors were horizontal and recipients were pendant), it is possible that bees visited pendant flowers fewer times, resulting in lower pollen deposition. Bees can also learn to associate visual cues with pollen presence (Muth et al., 2016), which could extend to floral orientation, thus enabling bees to learn to choose or avoid flowers based on orientation. In our experiment, the relatively short time frame of each trial may not be sufficient to elicit bumblebee learning of orientations, although we did not quantify how visitation varies through time. In any case, it is likely that visitation rates to recipients and donors remain similar when all flowers are pendant, and bees have no alternative flowers from which to choose. In this case, factors other than pollinator choice, for instance, pollinator positioning on the flower, are needed to explain why we observe reduced receipt of outcross pollen in *S. rostratum*. In summary, changes in pollinator behavior may mediate the effect of floral orientation on pollen transfer, but further behavioral observations and separate quantification of self and outcross pollen are required.

## CONCLUSIONS

Our study suggests that floral orientation and bilateral symmetry interact to affect outcross‐pollen transfer in buzz‐pollinated plants, therefore supporting the general importance of floral orientation for pollen transfer across systems with different animal pollinators. Although pollen release in buzz‐pollinated flowers appears to be equally effective regardless of the orientation of the anthers, pollen transfer to the pollinator body and receipt in the stigma is likely mediated by alignment and fit of the pollinator and flower, which is influenced by floral orientation in bilaterally symmetric flowers. A horizontal orientation may thus have evolved to promote outcrossing in bilaterally symmetric (heterantherous) flowers by obliging bees to adopt and repeat specific positions and alignment with the flower. In contrast, the orientation of radially symmetrical flowers may be under fewer selective constraints regarding outcross‐pollen transfer by animal vectors. Even though many radially symmetric, buzz‐pollinated flowers are pendant, the selective advantages of this orientation remain largely unexplored. Further studies are required to assess the consequences of the interaction between floral orientation and symmetry across different types of flowers and pollinators, but our findings suggest that floral orientation is key for the cross pollination of plants with bilaterally symmetric, complex flowers, and ultimately for their evolution.

## AUTHOR CONTRIBUTIONS

L.N. designed and performed the experiments and conducted the analyses. M.V.M. supervised the research, and L.N. and M.V.M wrote the manuscript.

## Supporting information


**Appendix S1**. *Solanum* material used in this study.

## Data Availability

The data set and code generated and analyzed in this study are available at the University of Stirling's DataStorre repository: http://hdl.handle.net/11667/204.
