# Supplementary material for: Floral orientation affects outcross‐pollen deposition in buzz‐pollinated flowers with bilateral symmetry
Source: Am J Bot. 2022 Oct 17;109(10):1568–78. doi: 10.1002/ajb2.16078 (PMC9828177; doi:10.1002/ajb2.16078)
Supplement: Supplementary file 1 — Appendix S1. Solanum material used in this study. [file AJB2-109-1568-s001.docx]

| Species | Accession | Latitude (N) | Longitude (W) | Population |
| --- | --- | --- | --- | --- |
| *Solanum rostratum* | 10-s-73 | \| 20.90 \| \| --- \| | 100.71 | San Miguel |
| *S. rostratum* | 10-s-74 | \| 20.90 \| \| --- \| | 100.71 | San Miguel |
| *S. seaforthianum* | 19-s-01 | 20.84 | 89.44 | Matilde |

Nevard and Vallejo-Marín – *American Journal of Botany* 2022 – Appendix S1

Appendix S1. Plant material of collected *Solanum* used in this study.
